# Novel FAP ligands enable improved imaging contrast in sarcoma patients due to FAPI-PET/CT

**DOI:** 10.1007/s00259-021-05374-4

**Published:** 2021-05-21

**Authors:** Stefan A. Koerber, R. Finck, K. Dendl, M. Uhl, T. Lindner, C. Kratochwil, M. Röhrich, H. Rathke, G. Ungerechts, S. Adeberg, K. Herfarth, D. Jaeger, J. Debus, U. Haberkorn, F. L. Giesel

**Affiliations:** 1 Department of Radiation Oncology, Heidelberg University Hospital, Im Neuenheimer Feld 400, 69120 Heidelberg, Germany; 2 Heidelberg Institute of Radiation Oncology (HIRO), Heidelberg, Germany; 3 National Center for Tumor Diseases (NCT), Heidelberg, Germany; 4 Department of Nuclear Medicine, Heidelberg University Hospital, Heidelberg, Germany; 5 Department of Radiation Oncology, Klinikum Ludwigshafen, Ludwigshafen, Germany; 6 Department of Medical Oncology, Heidelberg University Hospital and National Center for Tumor Diseases (NCT), Heidelberg, Germany; 7 Heidelberg Ion-Beam Therapy Center (HIT), Department of Radiation Oncology, Heidelberg University Hospital, Heidelberg, Germany; 8 German Cancer Consortium (DKTK), partner site Heidelberg, Heidelberg, Germany; 9 Clinical Cooperation Unit Radiation Oncology, German Cancer Research Center (DKFZ), Heidelberg, Germany; 10 Clinical Cooperation Unit Nuclear Medicine, German Cancer Research Center (DKFZ), Heidelberg, Germany; 11 Translational Lung Research Center Heidelberg (TLRC), German Center for Lung Research (DZL), Heidelberg, Germany; 12 Department of Nuclear Medicine, University Hospital Düsseldorf, Düsseldorf, Germany

**Keywords:** FAPI, PET, Sarcoma, Fibroblast activation protein, SUV

## Abstract

**Purpose:**

A high expression of fibroblast activation protein (FAP) was observed in multiple sarcomas, indicating an enormous potential for PET/CT using ^68^Ga-radiolabeled inhibitors of FAP (FAPI). Therefore, this retrospective study aimed to evaluate the role of the novel hybrid imaging probe for sarcomas as a first clinical evaluation.

**Methods:**

A cohort of 15 patients underwent ^68^Ga-FAPI-PET/CT for staging or restaging. The acquisition of PET scans was performed 60 min after administration of 127 to 308 MBq of the tracer. The uptake of ^68^Ga-FAPI in malignant tissue as well as in healthy organs was quantified by standardized uptake values SUVmean and SUVmax.

**Results:**

Excellent tumor-to-background ratios (> 7) could be achieved due to low background activity and high SUVmax in primary tumors (median 7.16), local relapses (median 11.47), and metastases (median 6.29). The highest uptake was found for liposarcomas and high-grade disease (range 18.86–33.61). A high SUVmax (> 10) was observed for clinically more aggressive disease.

**Conclusion:**

These preliminary findings suggest a high potential for the clinical use of ^68^Ga-FAPI-PET/CT for patients diagnosed with sarcoma.

## Introduction

Nowadays, positron emission tomography (PET)/computed tomography (CT) is widely used for clinical cancer imaging. For many malignant tumors, ^18^F-fluorodeoxyglucose (^18^F-FDG) is highly suitable as a PET tracer. Although sarcomas are uncommon, the use of ^18^F-FDG-PET/CT for the detection, staging, and oncological management of sarcomas has increased considerably, leading to improved target volume delineation for radiooncological treatment approaches as well as evaluation of treatment response [1–4]. Moreover, ^18^F-FDG-PET/CT provides a useful predictive tool for patients with soft tissue and bone sarcoma. Due to a high mitotic count sustained by intense glycolytic activity, especially high-grade soft tissue sarcomas can be well displayed by hybrid imaging using ^18^F-FDG tracer [5]. Although ^18^F-FDG-PET/CT represents a highly efficient staging and restaging probe for some sarcoma like rhabdomyosarcoma, physiological uptake within the brain limits the diagnostic value of the tracer, e.g., in the head and neck area [5].

Since the recent development of specific tracers that function as fibroblast activation protein inhibitors (FAPI) for hybrid imaging [6, 7], several studies demonstrated promising results with regard to imaging and tumor characterization. Although ^68^Ga-FAPI-PET/CT also has a high potential for benign diseases [8], its main use continues to be in the imaging of cancers. Kratochwil et al. observed a very high average SUVmax of more than 12 in sarcomas [9]. This is of great interest due to the low uptake of ^18^F-fluorodeoxyglucose (FDG) in low-grade sarcoma, demonstrating one of the numerous limitations of conventional FDG-PET/CT [10]. Historically, fibroblast activation protein (FAP) expression was initially described by Rettig et al. in 1988 and was found in malignant cells of many sarcomas [11–13]. Thus, sarcomas seem to be ideal candidates for using FAPI ligands as a staging probe, for tumor characterization, or even radioligand therapy. Therefore, this retrospective analysis aimed to characterize ^68^Ga-FAPI uptake in a cohort of 15 patients with different sarcomas.

## Materials and methods

### Patient cohort

We retrospectively analyzed 15 patients (6 females, 9 males) with the diagnosis of sarcoma. All patients gave written informed consent to undergo ^68^Ga-FAPI-PET/CT on an individual patient basis following national regulations and the Declaration of Helsinki. The radiopharmaceutical was synthesized and labeled according to the German Pharmaceutical Act §13(2b) (approval of the local ethical committee S016/2018). All patients were referred for experimental diagnostics by their treating oncologist supplementing standard diagnostic imaging. Examples of indications are inconclusive findings or insufficient tumor delineations. Four out of 15 patients were already part of the initial analysis performed by Kratochwil and colleagues [9].

### Radiopharmaceuticals and ^68^Ga-FAPI-PET/CT imaging

Chemical synthesis and labeling of ^68^Ga-FAPI-04 (6), ^68^Ga-FAPI-46 (5), and ^68^Ga-FAPI-74 (4) followed the methods as described in previous publications [6, 7, 14]. The radiopharmaceutical was administered intravenously followed by image acquisition 60 min after tracer application. The injected activity ranged from 127 to 308 MBq (2–3 MBq per kg bodyweight). Patients were requested to self-report any new symptoms or abnormalities up to 30 min after the end of the examination. PET imaging was performed with a Biograph mCT Flow PET/CT Scanner (Siemens Medical Solutions). PET scans were conducted according to previously published protocols [9, 15]. Consequently, a low-dose CT without contrast was performed, followed by PET scans in 3-dimensional mode (matrix, 200 × 200). The emission data were corrected for random, scatter, and decay and subsequently reconstructed.

### Image evaluation

The tracer biodistribution in all patients was quantified by mean and maximum standardized uptake values (SUVmean and SUVmax), at 1 h after application. Calculating the SUV, circular regions of interest were drawn around the tumors on transaxial slices and automatically adapted to a 3-dimensional VOI with e.soft software (Siemens) at a 60% isocontour. Evaluation of normal organs was conducted with a 1-cm diameter (for the small organs, thyroid, parotid gland, myocardium, oral mucosa, spinal cord) or 2-cm diameter (the brain, muscle, liver, pancreas, spleen, kidney, fat, aortic lumen content, lung) sphere placed inside the organ parenchyma. The ^68^Ga-FAPI-PET/CT scans were analyzed by one board-certified radiologist, one board-certified radiation oncologist, and two board-certified nuclear medicine physicians in consensus. For the determination of tumor-to-background analyses (TBR), the geometrical mean was used. In defining the SUVs, median and range were utilized.

### Statistical analyses

Descriptive analyses of patients and their clinical and tumor-specific characteristics were performed.

All statistical analyses were conducted utilizing Excel (version 16.43 Microsoft) for Mac (Apple) and IBM SPSS Statistics 23.

## Results

The patients included in the current analysis had a median age of 61 years. Our cohort consisted of several types of sarcoma including five liposarcomas, three undifferentiated pleomorphic sarcomas (UPS), and two leiomyosarcomas (Table 1). 8 out of 15 patients (53.3%) had metastatic disease and local relapse was suspected in three patients. 2 out of the 8 patients with metastatic disease were already metastatic at first diagnosis. Most patients were diagnosed with low-grade sarcoma (80%). For three patients, the grading was not known. The time interval between the initial diagnosis and ^68^Ga-FAPI-PET/CT ranged from 1 to 5 months.

**Table 1:** Patient characteristics

Total patients	*n* = 15
Median age	61
Median MBq	261 (127–308)
Sex	
Female	6
Male	9
Tumor stage	
Metastatic disease	8
Local relapse	4
Other	3
Grading	
Low grade	8
High grade	4
Unknown	3
Histology	
Liposarcoma	5
UPS	3
Leiomyosarcoma	2
Ewing sarcoma	1
Osteosarcoma	1
Synovial sarcoma	1
Sarcoma NOS	1

*UPS*, undifferentiated pleomorphic sarcoma; *NOS*, not otherwise specified

**Fig. 1:**
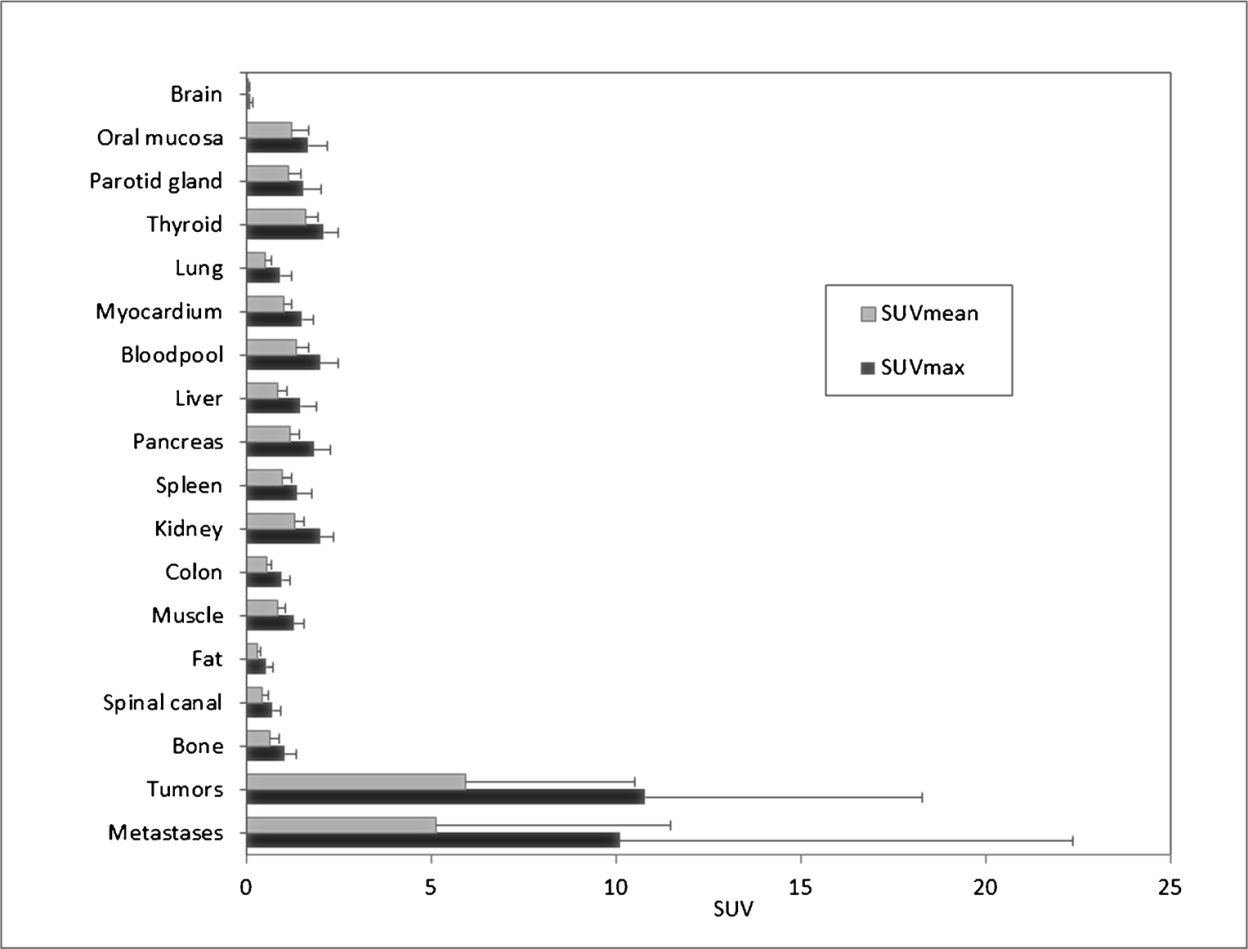
PET-based biodistribution analysis of 15 patients with ^68^Ga-FAPI-PET, imaged 1 h after injection

There was a very low background activity with an average normal organ uptake of 2.00 (SUVmax) and 1.34 (SUVmean) for bloodpool; 1.48 (SUVmax) and 0.86 (SUVmean) for normal liver parenchyma; 0.92 (SUVmax) and 0.53 (SUVmean) for normal lung parenchyma; and 1.50 (SUVmax) and 1.02 (SUVmean) for myocardium. ^68^Ga-FAPI-PET/CT detected four primary tumors with a median SUVmax and SUVmean of 7.16 (range 4.64–9.79) and 3.27 (range 2.28–5.51) as well as three local relapses with a median SUVmax and SUVmean of 11.47 (range 6.35–26.24) and 7.03 (range 3.00–13.08), respectively. For metastases, median SUVmax and median SUVmean of 6.29 (range 2.06–37.25) and 3.01 (range 1.45–19.44) were obtained, while the highest uptake was found for lung metastases (median SUVmax: 37.35; median SUVmean: 19.44). Thus, excellent tumor-to-background ratios of more than 7.00 (tumor-to-bloodpool, SUVmax and SUVmean) could be achieved (Fig. 1).

**Fig. 2:**
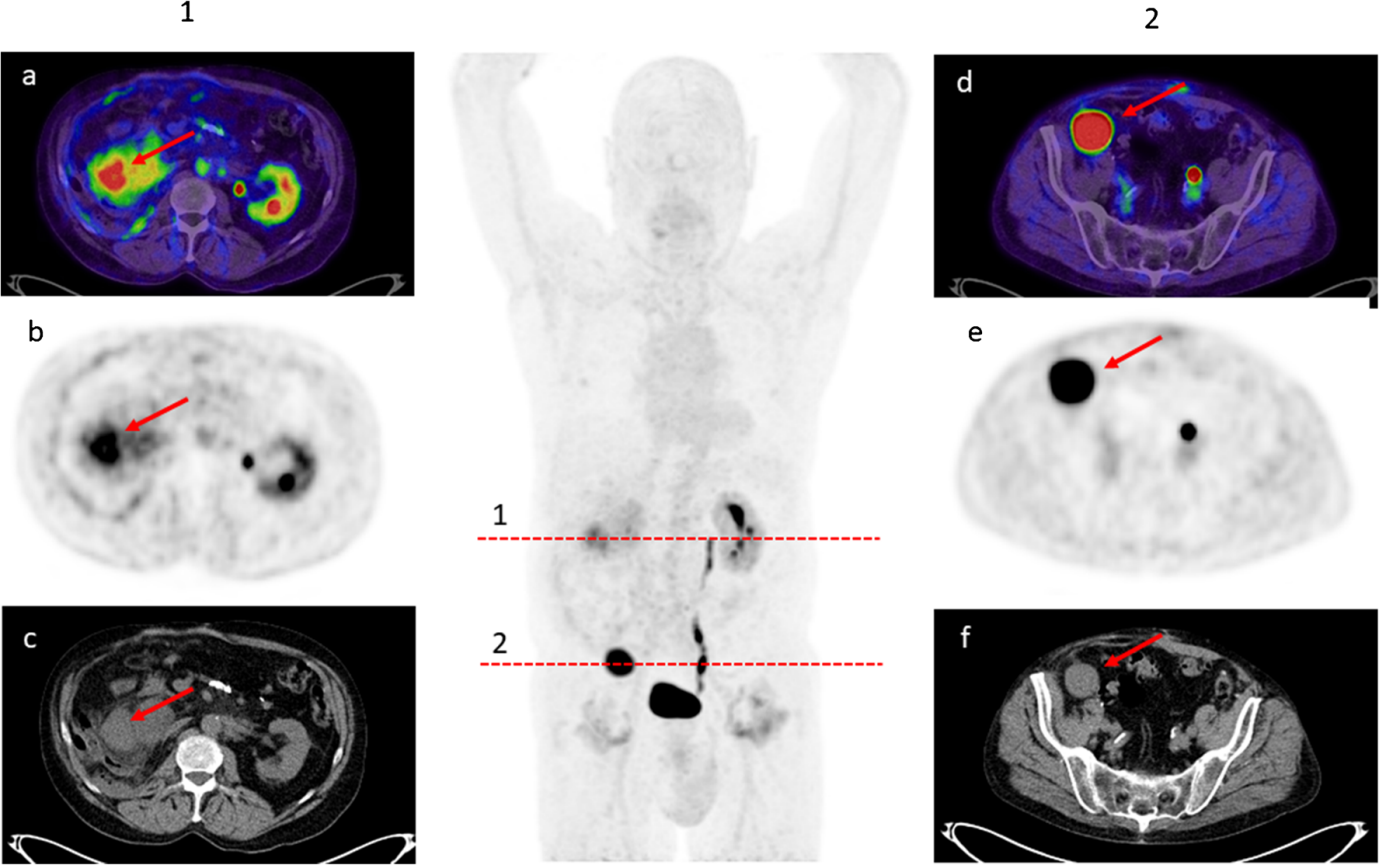
69-year-old patient with a bi-focal relapse of a liposarcoma detected by ^68^Ga-FAPI PET/CT (**a**, **d** FAPI PET/CT Dx; **b**, **e** FAPI-PET Dx; **c**, **f** CT Dx). (1) SUV_max_ 8.98 SUV_mean_ 4.60 (2) SUV_max_ 19.39 SUV_mean_ 13.18

In subgroup analyses with regard to histology, the highest uptake was found for liposarcomas with a median SUVmax and SUVmean of 14.19 and 8.90, respectively. Moreover, ^68^Ga-FAPI uptake was higher for high-grade sarcomas compared to low-grade disease (median SUVmax: 30.21 (range 9.79–44.29) vs. 6.35 (1.89–14.05); median SUVmean: 16.49 (5.91–22.39) vs. 2.90 (1.2–10.01)) (Table 2; Fig. 2).

**Table 2:** ^68^Ga-FAPI uptake according to histological type and grading

Diagnosis	*n*	Grading	SUVmax (lesions)	Range	SUVmean (lesions)	Range	*n* (metastases)
UPS	3	High grade	30.21	9.79–44.29	16.61	5.91–22.39	4
Liposarcoma	1	High grade	26.24	18.86–33.61	14.87	11.46–18.27	/
	4	Low grade	7.59	4.64–14.05	3.56	2.28–10.01	1
Myxofibrosarcoma	1	Low grade	6.35	6.35	3.00	3.00	/
Leiomyosarcoma	2	Low grade	6.56	3.85–8.10	2.80	2.66–4.63	7
Synovial sarcoma	1	Low grade	2.06	1.89–2.23	1.45	1.20–1.69	2
Ewing sarcoma	1	Unknown	7.90	6.5–15.15	3.28	2.90–4.66	3
Sarcoma NOS	1	Unknown	7.09	7.09	3.21	3.21	/
Osteosarcoma	1	Unknown	3.16	2.91–3.86	1.88	1.84–2.14	6
	4	High grade	30.21	9.79–44.29	16.49	5.91–22.39	4
	8	Low grade	6.35	1.89–14.05	2.90	1.2–10.01	10

*UPS*, undifferentiated pleomorphic sarcoma; *NOS*, not otherwise specified

## Discussion

The current analysis reveals a high intratumoral ^68^Ga-FAPI uptake for sarcomas and sarcoma metastases, indicating a promising clinical application of FAPI-PET/CT for this tumor entity. Compared to data obtained from conventional PET imaging, standardized uptake values probably reached higher levels than using ^18^F-FDG tracer: A preliminary report from a prospective trial evaluating the SUVmax of benign and malignant soft tissue lesions for patients undergoing^18^F-FDG-PET/CT resulted in a SUV range of 4.1–18.1 for sarcomas [16]. In a histological subgroup analysis of a large retrospective audit of 957 consecutive ^18^F-FDG scans of 493 patients, the highest uptake was observed for UPS, angiosarcoma, and leiomyosarcoma (each mean SUVmax <20) [17]. For the current FAPI cohort, the highest SUVmax was obtained for UPS (median SUVmax: 30.21, mean SUVmax 28.10) and liposarcoma (median SUVmax: 26.24, mean SUVmax 26.24) (Table 2). For both, ^68^Ga-FAPI and ^18^F-FDG, a significantly higher SUV was found for high-grade sarcoma independent of histological subtype [17]. Moreover, both tracers demonstrated similar intralesional uptake in metastases: the mean SUVmax ranged from 3.56 to 6.16 for ^18^F-FDG, which is comparable to intrametastatic ^68^Ga-FAPI uptake (mean SUVmax: 10.5) [18]. Interestingly, Iagaru et al. reported on a very low FDG signal for lung metastases (mean SUVmax: 3.56), while for the current FAPI cohort, the highest uptake was observed for pulmonary lesions (mean SUVmax: 17.34) [18]. Confirmatory studies in this population are scarce; however, this various range of tracer uptake may be explained by different histological subtypes and/or grading as well as a possible increased activity of fibroblasts in the lung parenchyma.

Data obtained from histopathological studies are clearly consistent with our results. Dohi et al. reported on a consistently high expression of FAP in bone and soft tissue tumor cells analyzing various sarcoma types like osteosarcoma, Ewing sarcoma, and rhabdomyosarcoma [12]. In osteosarcoma cells, significant inhibition of cell proliferation, migration, and invasion was observed after a knockdown of the serine protease [19], representing the importance of FAP for the malignant behavior of these tumors after its upregulation. Hereby, FAP most probably affects tumor stage and prognosis as demonstrated in a recently published meta-analysis by Liu et al.: the authors observed that FAP expression was associated with an increased risk for nodal metastases, higher local tumor invasion, and poor survival [20]. Similar results were also obtained for sarcomas like osteosarcoma, for instance. A higher expression of the serine protease was associated with a high histological grade, a more advanced stage, presence of distant metastasis, and a worse progression-free and overall survival [21, 22].

Apart from a high tracer uptake in high-grade sarcoma, a high SUVmax of more than 10 was observed in four patients (2 patients with local relapse, 2 patients with multifocal metastatic disease), while only two of these patients were diagnosed with high-grade disease. After comparison with clinical data, both patients with local relapse showed aggressive courses of disease with multiple relapses (> 10). Although the subgroup was quite small, these results may indicate that ^68^Ga-FAPI-PET/CT might be able to additionally provide a rough assessment of the course of the disease considering a possibly increased presence of cancer-associated fibroblasts within that area.

**Fig. 3:**
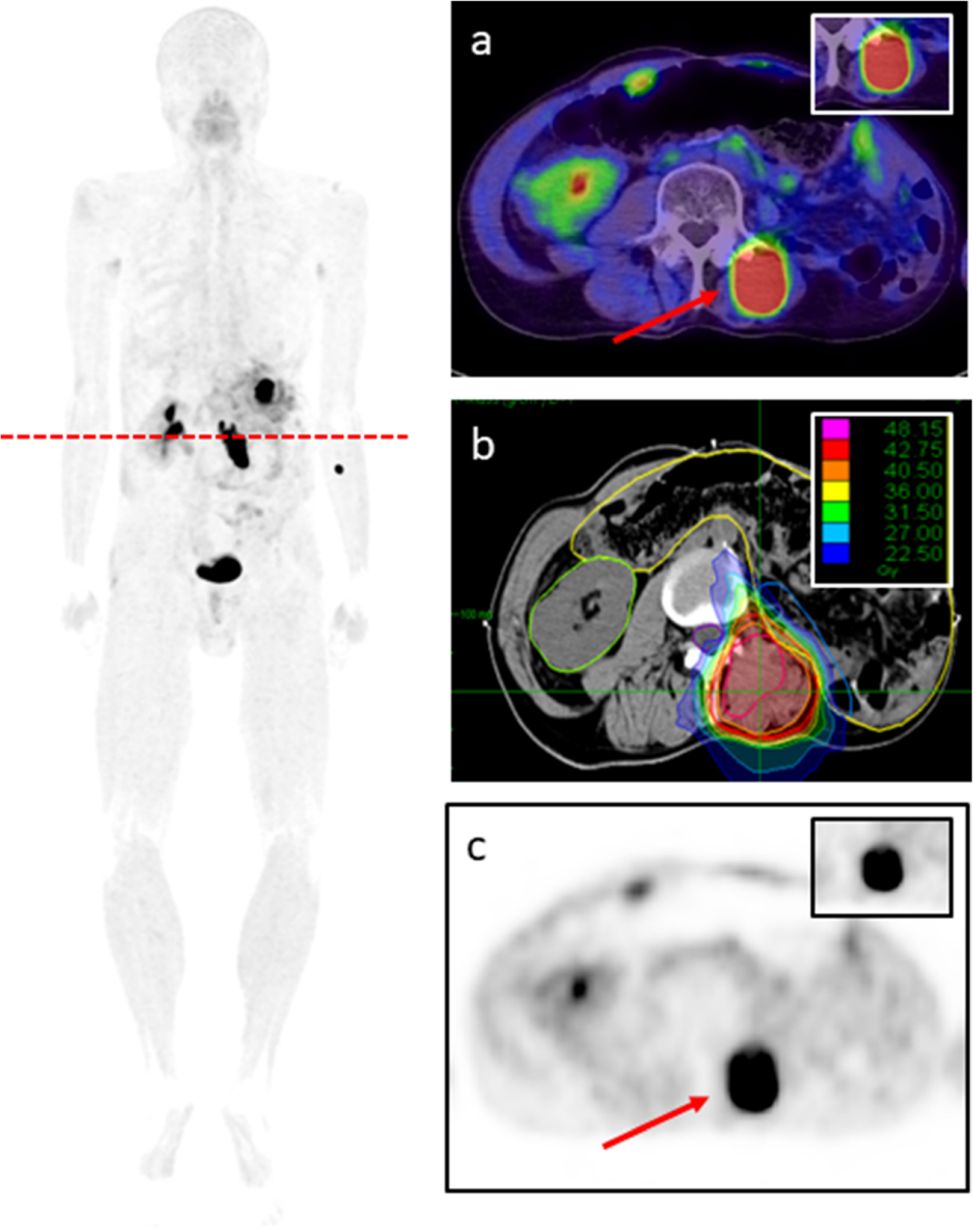
Local irradiation (**b**) of relapsed liposarcoma undergoing ^68^Ga-FAPI PET/CT (**a**, **c**). SUV_max_ 33.61 SUV_mean_ 18.27

Within the last years, some advances could be achieved for sarcoma patients due to improved surgery or innovative systemic treatment approaches; however, median overall survival rates range between 18 and 24 months for metastatic disease [23, 24]. Therefore, the current clinical situation and the histopathological background make sarcomas very suitable candidates for FAPI ligands. Apart from diagnostic imaging for staging or restaging, FAPI ligands can be used for radioligand therapy, potentially improving oncological outcomes [25]. Moreover, for all 4 patients undergoing radiotherapy, tumor volume delineation was improved due to an excellent FAPI positivity within the primary tumor or local relapse (Fig. 3).

To the best of our knowledge, this is the first clinical study evaluating ^68^Ga-FAPI uptake in a cohort of patients with sarcoma, although the number of patients included in this retrospective analysis was small and there is a lack of standard of reference to evaluate the performance of the novel tracer. Moreover, no patient received FDG-PET/CT which could be compared with the new hybrid imaging approach. However, the current study shows the potential of the new ligand as a diagnostic or even prognostic agent and may encourage prospective studies in the future.

## Conclusion

The current study demonstrated that ^68^Ga-FAPI-PET/CT is a very promising imaging probe for patients with sarcomas. This small case series suggests a high tracer uptake and excellent tumor-to-background ratios. FAPI ligands have the potential for improving the diagnostic and possibly the therapeutic approach for this malignancy; however, larger prospective studies will have to confirm the findings of this study.

## Data Availability

The data used and/or analyzed during the current study are available from the corresponding author on reasonable request.
